# Digital treatment for tinnitus combining tinnitus retraining therapy and cognitive behavioral therapy: a Norwegian naturalistic study

**DOI:** 10.3389/fpsyt.2026.1868527

**Published:** 2026-09-10

**Authors:** Katrine Sol Dalseng, Natalia Liavåg, Dag Lyngve Sunde, Stian Solem

**Affiliations:** 1 Department of Psychology, Norwegian University of Science and Technology, Trondheim, Norway; 2 Department of Ear, Nose, Throat, and Maxillofacial Surgery, Møre og Romsdal Hospital Trust, Molde, Norway

**Keywords:** anxiety, depression, digital treatment, hyperacusis, ICBT, misophonia, THI, tinnitus

## Abstract

**Introduction:**

Ten to fifteen percent of the population experience tinnitus. It is necessary to develop effective treatments for patients suffering from tinnitus. This study aimed to explore the effectiveness of an internet-based treatment consisting of tinnitus retraining therapy and cognitive behavioral therapy for patients with primarily tinnitus. The secondary aims were to investigate changes in hyperacusis (hypersensitivity to sounds), misophonia (strong emotional reaction to a sound), depression, and anxiety.

**Methods:**

A total of 144 patients diagnosed with tinnitus were included and assessed before treatment, and after 90 and 180 days. The results were differentiated for patients with mild, moderate, and severe+ tinnitus according to their scores on the Tinnitus Handicap Inventory (THI).

**Results:**

A significant change in scores on the THI (*d* = 0.41) was found from pre-treatment to the 180-day assessment for the total sample. A large effect size (*d* = 0.95) was found for the group with severe+ tinnitus and a small effect size for the group with mild tinnitus (*d* = 0.22). Further, there were small improvements in anxiety, depression, and hyperacusis, but no change in misophonia.

**Discussion:**

In conclusion, this study indicate that the treatment can be helpful for people with tinnitus.

## Introduction

The term tinnitus is a condition defined as a subjectively perceived sound in the head or ears without the presence of external sound (1). Common sound descriptions among people with tinnitus include ringing, hissing, cicadas, running water, and the grinding of steel (2). Higher severity of tinnitus is associated with higher rates of anxiety, depression, heightened stress responses, concentration problems, and insomnia (3).

Several behavioral and educational interventions have been developed to address tinnitus distress. Among these, Tinnitus Retraining therapy (TRT) is one treatment option, combining educational counselling and sound therapy as its core components (4). Sound therapy is suggested to be used with low-level neutral auditory signals to facilitate the habituation of tinnitus (5). The aim of TRT is for the individual to habituate to the tinnitus, thereby reducing the attentional resources allocated to it. A systematic review from 2025 including 2069 patients found that while TRT is effective in reducing tinnitus distress, other treatment options, such as Cognitive Behaviour Therapy (CBT), may be equally or more effective, but also that combining TRT with CBT may enhance overall treatment efficacy (6). Cima et al. (7) developed a treatment approach combining TRT and CBT and found significant improvements in tinnitus severity and health-related quality of life. Similarly, another study tested the effects of TRT followed by CBT and found reductions in anxiety, depression, hyperacusis, and perceived tinnitus loudness (8). There are no existing Norwegian treatment guidelines for tinnitus, but the National Institute for Health and Care Excellence (9) guideline recommends CBT and to further research the combination of TRT with tinnitus support. A review of guidelines for tinnitus stated that greatest variation occurs in recommendations regarding therapies involving sound (e.g. TRT) (10).

CBT has also been developed to modify psychological processes that contribute to or sustain tinnitus distress (11). CBT is shown to be helpful for reducing the patient’s annoyance of tinnitus and alleviating symptoms associated with the condition (12). The goal of CBT is to modify dysfunctional thoughts and maladaptive coping strategies, thereby altering the patient’s emotional reaction toward their tinnitus. Hesser et al. (12) conducted a systematic review and meta-analysis of 15 randomized controlled trials of CBT for tinnitus distress in 2011, including 1091 patients. They found that CBT significantly reduces tinnitus distress, with moderate to large effect sizes of Hedges’s *g* = 0.70 (vs. waiting list) and *g* = 0.44 (vs. education or other treatments).

While CBT is an established treatment for tinnitus there are also challenges as reviewed by Hedman et al. (13). The review suggested; 1) that access to treatment remains limited, 2) there is a shortage of trained therapists, 3) substantial time and financial resources are required, and 4) in rural areas, long travel distances to clinics further reduce the practicality of traditional CBT. In recent years, the development of digitalized treatment modalities has extended to CBT, giving rise to Internet based CBT (ICBT). This approach combines digitally delivered bibliotherapy with therapist interaction conducted online. Patients receive the same core components as traditional CBT, but psychoeducational materials and behavioral instructions are delivered using an online platform (13). A meta-analysis found that internet-based interventions like ICBT for tinnitus showed a medium effect size (*d* = 0.59) (14).

Patients with severe tinnitus tend to be more frequently diagnosed with anxiety disorders than those with milder tinnitus (15). A meta-analysis of several internet-based psychological treatments for tinnitus found a small to moderate effect size on anxiety (*d* = 0.37) (14). Depressive disorders are also frequently diagnosed among patients with tinnitus, and depressive symptoms can significantly worsen their tinnitus distress (15). ICBT for tinnitus seems to have a small effect on depression (*SMD *= 0.28 ± 0.07) (16).

Tinnitus also frequently co-occurs with hyperacusis, a condition up to 25% of patients with tinnitus have (5). Hyperacusis is a hypersensitivity to sound, where the individual perceives sounds as abnormally loud and has an exaggerated response to them (17). What distinguishes hyperacusis from simple annoyance to loud sounds is that the sounds provoking an exaggerated response are not uncomfortably high or threatening. The sounds themselves are typically low in intensity, yet individuals with hyperacusis may react strongly to everyday sounds across a wide range of volumes (17). A randomized controlled trial from 2018 examining the effect of ICBT on tinnitus found that treatment also reduced symptoms of hyperacusis, with a between-group effect size of 0.3 when compared to a passive control group (18).

Misophonia also shares significant commonalities with tinnitus (19). A study found that 42% of patients seeking help for tinnitus also exhibited symptoms of misophonia (20). Misophonia is defined as “an abnormally strong reaction occurring to a sound with a specific pattern and/or meaning to an individual” (21, p.106). Hyperacusis and misophonia are distinct forms of decreased sound tolerance: hyperacusis is associated with physical discomfort or pain elicited by sounds that exceed an individual’s lowered loudness discomfort level, while misophonia involves intense emotional reactions to particular trigger sounds regardless of their loudness (22). With misophonia, the strong emotional response to a specific sound often creates feelings of anger, hate, or disgust toward the individual or object making that sound (23). The specific sound that triggers misophonia varies, the most common sounds are chewing, swallowing, or others breathing (23). Standard treatments for misophonia have typically involved approaches used for tinnitus, such as TRT and CBT (19). To this date there is limited research of treatment options for misophonia, but a systematic review from 2023 pointed out CBT as the most promising one (24).

While the effect of ICBT on tinnitus is well documented, no prior studies have investigated ICBT or TRT within a Norwegian population. Furthermore, most of the studies follow a manual based on Andersson’s model of ICBT (11). Our approach differs from this model by integrating standard CBT modules with elements from TRT. This hybrid approach mirrors the face-to-face treatment developed by Cima et al. (7) and addresses recent recommendations by Alashram (6). To our knowledge, no research has yet evaluated the combination of CBT and TRT using a digital format.

Building on previous research and the growing need for accessible treatment options, this study examines the effectiveness of a digital treatment program for individuals with tinnitus. The primary aim is to investigate whether digital treatment reduces tinnitus symptoms, as well as comorbid conditions such as misophonia, hyperacusis, anxiety, and depression. The main hypothesis is that treatment would be associated with a medium to large effect size for tinnitus symptoms, and a small effect size for depression, anxiety, and hyperacusis (14, 18). However, research suggests that patients with greater tinnitus severity benefit most from treatment (25, 26), but treatment trials and meta-analyses have not usually reported effect sizes differentiated according to tinnitus severity. Consequently, these studies support the argument for analyzing tinnitus based on symptom severity, and we expect that effect sizes could be larger for patients with higher tinnitus severity. Finally, although there is limited empirical evidence available for treatment of misophonia, CBT has been mentioned as a promising intervention, therefore we expected improvement in misophonia symptoms.

## Methods

This study is a single arm, prospective observational study without a control group. The study was registered and approved at Regional Committees for Medical and Health Research Ethics (ref.nr. 771693), and the hospital’s Data Access Committee (ref.nr. 2024/3997). All participants provided voluntary consent to participate in the study. Before initiating the treatment used in this study, patients were invited to participate, and only those who provided informed consent were included in the study. The patients were referred because of tinnitus complaints by their general practitioner and then examined and assessed by an ear, nose, and throat (ENT) specialist. Out of 424 referred patients, a total of 294 (69%) gave their consent to be included in the study.

### Participants and procedure

To be included in the study the patients had to: a) be diagnosed with tinnitus, b) be between 18 and 90 years of age, and c) give their consent to the study. If patients had vestibular schwannoma or other vestibular disorders, they were excluded from the study. There was no exclusion criterion based on other comorbidities. Initially there were 294 consenting patients, but 29 with slight to no tinnitus handicap according to Tinnitus Handicap Inventory (THI; scores below 16) were excluded because of subclinical symptoms. Another 121 patients were excluded because they did not answer questionnaires before starting the digital treatment or after 90 days. The final sample therefore consisted of 144 patients. Participant demographics for the 144 patients in our study are presented in **Table 1**.

**Table 1:** Participant demographics and clinical profile at pre-treatment (N = 144).

		1. Mild (*n* = 47)	2. Moderate (*n* = 59)	3. Severe+ (*n* = 38)	
Variable	Item	*N (%)*			*p*
Gender	Male	24 (51.06)	27 (45.76)	22 (57.90)	ns.
Age					ns.
	18-30	1 (2.13)	1 (1.69)	2 (5.26)	
	31-40	2 (4.26)	8 (13.56)	3 (7.89)	
	41-50	10 (21.28)	11 (18.64)	8 (21.05)	
	51-60	21 (44.68)	25 (42.37)	18 (47.37)	
	61-70	9 (19.15)	9 (15.25)	5 (13.16)	
	71+	4 (8.51)	5 (8.47)	2 (5.26)	
Employment					ns.
	Working	32 (68.09)	37 (62.71)	23 (60.53)	
	Student	0 (0.00)	2 (3.39)	1 (2.63)	
	Disability	15 (31.91)	20 (33.90)	14 (36.84)	
Hearing aids	Yes	13 (27.66)	25 (42.37)	17 (44.74)	ns.
Years of tinnitus duration					ns.
	< 1	5 (10.64)	4 (6.78)	4 (10.53)	
	1 - < 2	11 (23.40)	7 (11.86)	8 (21.05)	
	2 - < 4	8 (17.02)	14 (23.72)	6 (15.79)	
	4+	23 (48.94)	34 (57.62)	20 (52.63)	
Hyperacusis	Yes	4 (8.51)	13 (22.03)	7 (18.42)	ns.
Misophonia	Yes	26 (55.32)	40 (67.80)	24 (63.16)	ns.
M (SD)					
THI		27.49 (5.59)	46.68 (5.22)	72.95 (10.59)	1 < 2 < 3
HQ		15.60 (7.34)	20.39 (7.25)	20.89 (8.43)	1 < 2, 3
MQ		16.66 (11.72)	20.58 (11.44)	21.37 (14.35)	ns.
PHQ-9		5.68 (3.82)	8.83 (4.62)	11.87 (5.42)	1 < 2, 3
GAD-7		3.62 (3.42)	6.58 (4.10)	9.82 (5.47)	1 < 2, 3

THI, Tinnitus Handicap Inventory; MQ, Misophonia Questionnaire; HQ, Hyperacusis Questionnaire; PHQ-9, Patient Health Questionnaire-9; GAD-7, General Anxiety Disorder-7. Hyperacusis, HQ scores range from 0 to 42. Misophonia, MQ scores from 15 to 68. The three severity groups are categorized according to THI scores: Mild (18-36), Moderate (38-56), and Severe+ (58-100).

All patients considered for treatment had been referred to an ENT specialist for a standardized examination. This included a standard ENT examination containing anterior and posterior rhinoscopy, examination of collum and otomicroscopy, and four audiometry tests: tympanometry, pure tone audiometry, speech test, and threshold of discomfort measurement. If otomicroscopy or audiometric results indicated potential medical abnormalities, like unilateral sensorineural hearing loss or pulsatile tinnitus, an MRI of the temporal bone was performed to exclude underlying conditions that might explain the tinnitus. Any type of tinnitus was included in the study, except if tinnitus was caused by vestibular schwannoma. Data on prior history of tinnitus treatments was not recorded, but patients’ use of hearing aids was registered. The patients received no other tinnitus treatment at Norwegian hospitals while undergoing this treatment.

### Measures

There were dispatched five questionnaires before the patients started on the digital treatment, after 90 days, and after 180 days. The questionnaire interval was chosen because it was similar to previous studies (7, 8, 18) and allow easier comparisons. The patients started treatment right after the baseline questionnaires were obtained. The questionnaires were used to measure tinnitus severity, hyperacusis, misophonia, symptoms of depression and symptoms of anxiety. Answers to the questionnaires were collected using the same digital platform used for treatment delivery.

The Tinnitus Handicap Inventory (THI; 27, 28) measures the impact of tinnitus on daily living. THI consists of 25 items, and the global score can range from 0 to 100 and is categorized into five levels of tinnitus severity: slight-no handicap (0-16), mild handicap (18-36), moderate handicap (38-56), severe handicap (58-76), and catastrophic handicap (78-100).

The General Anxiety Disorder-7 (GAD-7; 29, 30) measures anxiety based on the past two weeks. It consists of seven items using a 0–3 rating scale giving a total score ranging from 0 to 21. The cut off scores are 0–5 for minimal anxiety, 6–10 for mild anxiety, 11–14 for moderate anxiety, and 15–21 for severe anxiety.

The Patient Health Questionnaire-9 (PHQ-9; 29, 31) is a 9-item self-report questionnaire used to assess severity of depression. The PHQ-9 uses a rating scale of 0-3, and scores between 20–27 indicate severe depression, 15–19 moderately severe, 10–14 moderate, 5–9 mild, and 0–4 minimal.

The Hyperacusis Questionnaire (HQ; 32, 33) is the most used clinical questionnaire to measure severity of hyperacusis. HQ consists of 14 items rated on a Likert scale ranging from 0 (never) to 3 (a lot). The total score ranges from 0–42 where a cutoff score of ≥ 28 indicates the presence of hyperacusis.

The Misophonia Questionnaire (MQ; 34, 35) measures symptoms of misophonia, how it affects emotion, behavior, and its severity. MQ consist of 17 questions rated on a Likert scale ranging from 0 (never) to 4 (always). The total score ranges from 0-68. Though higher scores indicate a higher level of misophonia, there is no official cut-off for the MQ, but some studies have used a score of 14 as a cut-off (36).

### Treatment

All patients followed the same treatment, consisting of an introduction seminar followed by a module-based internet treatment. The patients also had the opportunity to send a message to a therapist whenever they wanted throughout the treatment. All patients were offered the choice between a physical seminar or an internet-based seminar, the educational content was identical in both formats. The seminar was 6-hour long and contained information about the pathophysiology behind tinnitus, psychoeducation about the auditory system, the etiology, and common beliefs about tinnitus. Additionally, it provided an introduction to the internet-based treatment, focusing on the utility of TRT and CBT in coping with tinnitus. Completion of this seminar was a mandatory prerequisite for entering the digital treatment. Patients with misophonia or hyperacusis could also attend a separate webinar with content related to these issues. These webinars included education about misophonia/hyperacusis and education about desensitization towards hyperacusis and/or misophonia. These seminars had a duration of two hours, and the patients could sign up for further follow-up with an audiologist with certification in CBT if needed.

The treatment consists of components from both TRT and CBT. It was developed by an ENT specialist who specializes in tinnitus. The ENT specialist was not certified in CBT but was assisted by a certified CBT-psychologist. If the patients did not log onto the internet-based treatment, they received a weekly text message to remind them to follow the treatment.

The TRT consisted of 7 modules. These modules were represented using text and videos. These modules followed a standard TRT setup, with an overview of TRT, information about the auditory system and brain functions (patient education), pathophysiology, and the neurophysiological explanation model for tinnitus. Additionally, it included specific modules of sound therapy and reduced sound tolerance, like hyperacusis and misophonia. The module about sound therapy consisted of information about avoiding silence in general. Noise generators were not used as part of the treatment. The main goal of the TRT modules was to habituate the patient to their tinnitus. After finishing the TRT modules, the patients proceeded to the CBT modules. The modules were completed at the patient’s own pace.

The CBT consisted of 12 modules which included a short overview, Socratic questions, the cognitive diamond, sustaining factors, cognitive distortions, the ABC-model, set of rules the patients often live by, and alternative thoughts. With the ABC model, patients are taught that consequences (C) of an activating event (A) are mediated by the individual’s beliefs (B) of that event. These beliefs are challenged in CBT. The patients also had access to a personal behavior-diary, and digital worksheets where they could write down their own cognitive distortions regarding tinnitus and replace them. The patients also had the opportunity to attend group webinars led by a psychologist if they needed further help to develop their skills in CBT. The patients had access to the modules for 365 days after starting the treatment. The treatment encouraged patients to actively use what they had learned in the modules and make use of their personal behavioral-diary and digital worksheets after finishing the modules. **Supplementary Figure 1** illustrates elements of the digital treatment using six screenshots. Patients’ progress and time used on the different modules was not tracked.

### Statistical analysis

Due to the constantly ongoing admission process (enrollment was not study based but the treatment was implemented as usual care in the clinic), some consenting participants had not yet received all the questionnaires at the time of data collection. Of the 294 individuals who provided informed consent, 144 (49%) met the inclusion criteria and were retained for analysis. An intent-to-treat approach was used in the analyses. Among the 144 included patients, 46 (31.9%) participants had missing data for the 180-day follow-up questionnaires; these missing values were imputed using the Expectation-Maximization method. Little’s missing completely at random test indicated that the data were missing completely at random, χ²(142) = 112.27, *p* = .97.

Results were analysed for three severity groups: mild (*n* = 47), moderate (*n* = 59), and severe+ (*n* = 38). Potential differences between the three groups on questionnaire scores at pre-pretreatment were tested using Kruskal-Wallis tests (p-value set at *p* <.01). Chi-square tests were used for categorical variables.

Repeated measures analysis of variance was employed to assess changes in questionnaire responses across three time points: baseline, 90 days, and 180 days. The primary outcome measure was tinnitus severity (THI). The secondary outcome measures were assessed using questionnaires scores for anxiety, depression, hyperacusis, and misophonia. Tinnitus severity was included as a between-subject factor to investigate their potential moderating effects on treatment outcomes. Pairwise comparisons based on estimated marginal means adjusted for multiple comparisons were used to compare the three groups at the three timepoints and when changes in symptoms occurred. Assumptions of sphericity and Greenhouse Geisser were evaluated using Mauchly’s test. Effect sizes (Cohen’s *d*) were calculated using pooled standard deviations (*d* = *M*_1_ - *M*_2_/σ_pooled_). Effect sizes were interpreted as small (*d* = 0.2), medium (*d* = 0.5), or large (*d* = 0.8). The analyses were conducted using IBM SPSS (Statistical Package for the Social Sciences) version 31. Significance levels were set at *p* <.05.

## Results

Ten of the 144 patients were never registered as having accessed the digital treatment, but they completed the seminar and questionnaires. **Table 1** summarizes the demographic and clinical characteristics of the study population at pre-treatment. The sample comprised 144 patients (50.69% male), with the majority aged between 41 and 60 years. Most participants were employed (63.89%), and 61.81% did not use hearing aids. More than half (53.47%) had experienced tinnitus longer than four years. In terms of tinnitus severity, most patients reported mild to moderate levels of distress. Indications of hyperacusis was present in 16.67% of the sample, and 62.50% for misophonia.

There were no significant differences between the three groups on demographical variables. Based on mean scores, the mild and moderate tinnitus groups reported mild depression symptoms, while the severe+ group had moderate depression scores. The mild tinnitus group reported mild symptoms of anxiety, while the moderate and severe+ tinnitus groups had moderate anxiety symptoms. The mild group had less symptoms of hyperacusis, anxiety, and depression than the moderate and severe+ groups, while there were no differences in symptoms of misophonia. See **Supplementary Table 1** for an overview of Pearson’s correlations between the outcome measures.

**Figure 1:**
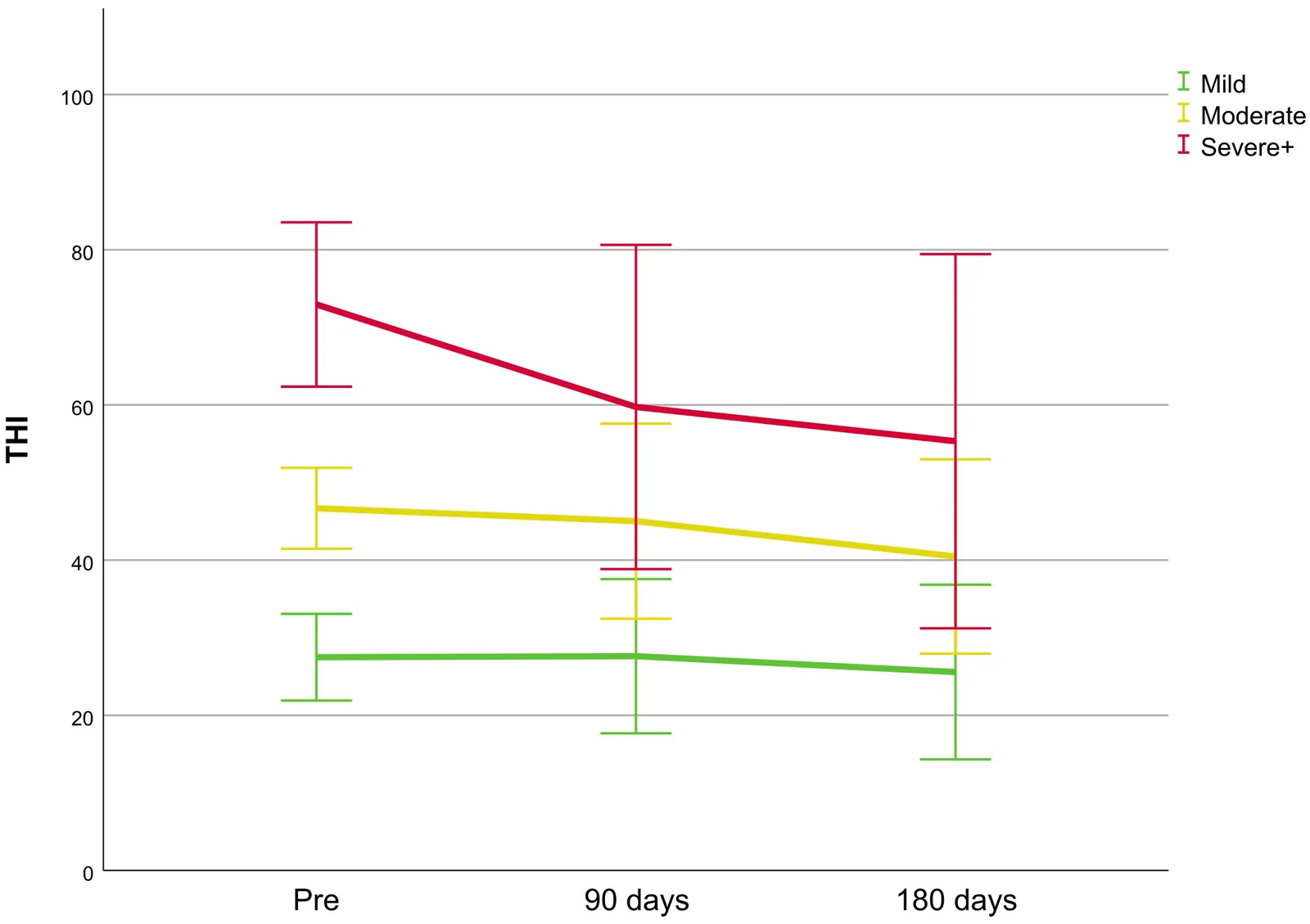
Differences in tinnitus severity following treatment (N = 144). THI, Tinnitus Handicap Inventory; mild handicap (18–36 points), moderate handicap (38–56 points), severe+ handicap (58–100 points). Error bars represent standard deviations. Central points represent mean THI scores.

**Table 2** summarizes the repeated measures ANOVA results for questionnaire scores over time. The patients reported lower THI scores after 180 days than they did before treatment. The effect size for the observed reduction in THI from pre-treatment to 180 days for the total sample was small-medium (*d* = 0.41). When comparing the non-imputed dataset with the imputed dataset, the effect sizes for THI were similar (*d* = 0.20 versus *d* = 0.22 after 90 days and *d* = 0.36 versus *d* = 0.41 after 180 days). For the severe+ group the effect size was large at 90 and 180 days, with *d* = 0.80 and 0.95 respectively. For the moderate group the effect size was small at 90 days (*d* = 0.17) and medium (*d* = 0.65) at 180 days. In contrast, for the mild group, the THI effect size was small, *d* = -0.02 (90 days) and 0.22 (180 days). In general, the reductions in tinnitus were greatest for the severe+ group, followed by the moderate and the mild group (see **Figure 1**).

**Table 2:** Changes over time in symptoms of tinnitus, hyperacusis, misophonia, anxiety, and depression.

Variable	Pre	90 days	180 days	*F*	Part Eta sq.	*d*	*d*
		*M(SD)*				90 days	180 days
THI							
Mild	27.49 (5.59)	27.62 (9.94)	25.57 (11.25)	9.61***	0.12	-.02	.22
Moderate	46.68 (5.22)	45.02 (12.57)	40.46 (12.53)			.17	.65
Severe+	72.95 (10.59)	59.74 (20.89)	55.32 (24.11)			.80	.95
Total	47.35 (18.82)	43.22 (19.04)	39.52 (19.63)			.22	.41
HQ							
Mild	15.60 (7.34)	15.81 (6.90)	14.65 (6.81)	0.90	0.01	-.03	.13
Moderate	20.39 (7.25)	19.42 (8.30)	17.59 (6.56)			.12	.40
Severe+	20.89 (8.43)	21.05 (8.00)	18.13 (7.86)			-.02	.34
Total	18.96 (7.91)	18.67 (8.02)	16.77 (7.11)			.04	.29
MQ							
Mild	16.66 (11.72)	15.23 (11.57)	16.11 (11.21)	0.21	0.00	.12	.05
Moderate	20.58 (11.44)	19.15 (11.77)	18.96 (11.90)			.12	.14
Severe+	21.37 (14.35)	20.37 (13.64)	20.31 (12.08)			.07	.08
Total	19.51 (12.43)	18.19 (12.32)	18.39 (11.77)			.11	.09
PHQ-9							
Mild	5.68 (3.82)	5.76 (4.00)	6.36 (3.76)	4.93**	0.07	-.02	-.18
Moderate	8.83 (4.62)	8.03 (4.45)	7.39 (3.89)			.18	.34
Severe+	11.87 (5.42)	10.11 (5.01)	9.17 (4.78)			.34	.53
Total	8.60 (5.16)	7.84 (4.74)	7.52 (4.22)			.15	.23
GAD-7							
Mild	3.62 (3.42)	4.29 (4.12)	4.21 (3.42)	4.82**	0.06	-.18	-.17
Moderate	6.58 (4.10)	5.93 (3.89)	5.80 (3.34)			.16	.21
Severe+	9.82 (5.47)	8.00 (4.88)	7.59 (5.07)			.35	.42
Total	6.47 (4.90)	5.94 (4.45)	5.75 (4.07)			.11	.16

THI, Tinnitus Handicap Inventory; MQ, Misophonia Questionnaire; HQ, Hyperacusis Questionnaire; PHQ-9, Patient Health Questionnaire-9; GAD-7, General Anxiety Disorder-7. *p = <.05, **p <.01, ***p <.001.

Pairwise comparisons on the THI indicated that the mild group experienced no significant change in tinnitus symptoms across the timepoints. The moderate group did not show any significant change from pre-treatment to 90 days, but the reduction from pre-treatment to 180 days was significant (*p* = .002). For the severe group there was a significant reduction in THI from pre-treatment to 90 days (*p* <. 001) and further reduction from 90 to 180 days (*p* = .004). All three groups’ mean scores on THI differed significantly at all timepoints (see **Figure 1**) as the severe+ group had highest score at all three assessments, followed by the moderate and mild groups, respectively.

On the HQ measure, the mild group showed no significant change in hyperacusis. The moderate group showed no significant change from pre-treatment to 90 days, but there was a significant reduction from pre-treatment to 180 days (*p* = .002) and from 90 days to 180 days (*p* = .021). The severe+ group showed no change from pre-treatment to 90-days, but there was a significant reduction from pre-treatment to 180 days (*p* = .014) and from 90 to 180 days (*p* = .003). The mild group had lower scores at all three timepoints compared to the two other groups, while there was no significant difference between the moderate and severe groups. On the MQ measure there were no significant changes in misophonia for the groups. Further, there were no differences in mean MQ scores at the three timepoints for the three groups. On the PHQ-9, the mild group showed no significant change in depressive symptoms. The moderate group showed a significant reduction from pre-treatment to 180 days (*p* = .003). The severe+ group showed a significant reduction from pre-treatment to 90 days (*p* = .010), from 90 to 180 days (*p* = .044), and from pre to 180 days (*p* <. 001). The mild group had lower scores at pre-treatment and 90 days compared to the two other groups, but at 180 days there was no significant difference compared with the moderate group. The severe+ group had significantly higher mean scores than the moderate group at all three timepoints.

On the GAD-7, the mild and moderate groups showed no significant change in anxiety symptoms. The severe+ group showed a reduction from pre-treatment to 90 days (*p* = .001) and from pre-treatment to 180 days (*p* <. 001). The mean scores on GAD-7 were different at all three timepoints for all three groups as the severe+ group had highest score at all three assessments, followed by the moderate and mild groups, respectively. The mild group had lowest scores across timepoints, the moderate group had higher scores than the mild group, and the severe group had higher scores than the moderate group. However, the difference between the moderate and severe group at 90 days was only borderline significant (*p* = .050).

## Discussion

Digital treatment combining TRT and CBT can be helpful for individuals suffering from tinnitus, and the reduction in symptoms was greater for individuals with more severe tinnitus distress. To our knowledge, this is the first study published focusing on digital psychological treatment for tinnitus tested on a Norwegian population, and this research provides preliminary evidence for the digital model combining both ICBT and TRT. The digital treatment of tinnitus showed a within-group effect size comparable to a previous similar meta-analysis (14), which is important for understanding and developing future tinnitus treatments. There were also significant reductions in the secondary measures (except for the mild tinnitus group) such as anxiety, depression, and hyperacusis, but not for misophonia. The main finding is consistent with previous research on combing TRT and CBT (7, 8) by showing reductions in tinnitus severity, anxiety, depression, and hyperacusis. Further, our study shows that this combination can also be delivered digitally. However, the study cannot conclude as to whether combining TRT with CBT is associated with any additional treatment gains.

The severe+ group demonstrated a large effect size (*d* = 0.95 at 180 days) in response to treatment, while the effect size for the mild group was small (*d* = 0.22 at 180 days). This finding of varying response to treatment depending upon disorder severity is consistent with previous research (25). In our sample, 32.6% had mild tinnitus, 41.0% moderate, and 26.4% severe+. The relatively low occurrence of severe tinnitus could raise questions about the sample’s level of dysfunction and why they seek help. However, the sample scored higher (*d* = 0.40) on baseline THI than in the Cima et al. (7) study (based on a comparison of means and standard deviations). If the THI cutoffs are valid, then it could be questioned why such a large part of the sample are classified as having mild tinnitus and why they are seeking treatment. One possible explanation is that they hope the tinnitus sounds will disappear. This is supported by a qualitative study on patient preferences in tinnitus treatment (37). They found that the patients initially hoped that their tinnitus would be cured, but later in the treatment process their focus changed to how to cope with their tinnitus. Regardless, the treatment could potentially have a preventive effect for the mild and moderate groups as they learn techniques that may prevent tinnitus symptoms from escalating. Further research is needed to determine this.

Evidence suggests that individuals with greater tinnitus severity may derive the greatest benefit from intervention (25, 26). Such findings should be interpreted with caution, as greater improvements among high-severity patients may partly reflect regression toward the mean and should ideally be controlled using a placebo condition as tinnitus symptoms may fluctuate (38). Patients with low baseline symptom severity often demonstrate smaller treatment-related changes, partly due to floor effects. A qualitative study could have yielded deeper insights into patients’ experiences of the treatment, as well as potential areas for improvement.

As a secondary outcome, we found a smaller effect size for anxiety symptoms (*d* = 0.16 for the total sample at 180 days) compared to what previous meta-analyses have found (small-moderate effect sizes) (14, 16). In our sample, the mean GAD-7 scores were relatively low, which could suggest potential floor effects. There was a larger effect size for the severe+ group (*d* = 0.42 at 180 days) than in the mild group (*d* = -0.17). This aligns with findings suggesting that anxiety symptoms are elevated among patients with more severe tinnitus (15). Similar findings were obtained regarding depression with a small effect size (*d* = 0.23 for the total sample) which is consistent with a meta-analysis (16).

The treatment was also associated with small improvements in hyperacusis (*d* = 0.29 for the total sample at 180 days). This finding is in line with Beukes et al. (18) who found that ICBT for tinnitus had an effect size of *d* = 0.3 for hyperacusis (2-months post-intervention). The difference in treatment effects between the severity groups was minimal, and the interaction effect was not significant. Jastreboff and Jastreboff (5) noted that up to 25% of individuals with tinnitus experience hyperacusis. In our study population, 17% had hyperacusis. These patients seemed to be distributed relatively evenly between the severity groups, which along with the low HQ scores, may account for the absence of an interaction effect. It is reasonable to suggest that ICBT targeting hyperacusis may yield larger improvements than those observed in our study. Supporting this, Jüris et al. (39) reported a large within-group effect size (*d* = 1.16 at post-treatment) using a CBT protocol tailored for hyperacusis.

Further, the findings indicate that the treatment was not associated with significant changes on misophonia. This lack of effect may be attributed to the fact that the treatment does not specifically target misophonia mechanisms, despite the overlap in standard treatment protocols for tinnitus and misophonia (19). In this study, a total of 62.5% of our participants scored above cutoff on the MQ. This could suggest that the cutoff value was set too low. The cutoff value was based on previous studies, but those studies set the threshold for detecting people who are sometimes sensitive to certain sounds (36). Further research is therefore needed on effective treatments for misophonia, but also to determine clinically meaningful cutoff scores for the MQ.

The findings from this study should be interpreted considering several limitations. First, the study had no control group. Without a control group, it is challenging to control for regression toward mean or ascertain anything about causality. Second, the available data did not capture information on treatment adherence, such as time spent engaging with the program, number of sessions or modules completed, or frequency of platform use. As a result, it remains uncertain how committed the patients were to the treatment, and whether participants completed the full intervention or only selected components. Consequently, the analyses cannot control for the effect of time spent with the modules which likely varied between participants. The only mandatory session was the seminar, and previous research has suggested that patients may benefit from a single session of tinnitus instruction and counselling (40). Early technical issues also allowed some participants to access all modules simultaneously rather than sequentially, which may have affected treatment engagement and outcomes. Furthermore, sound generators are typically used in TRT but was not used in this study.

A further limitation concerns the lack of data on supplementary therapeutic contact. It is unknown how many participants utilized additional support sessions with developers or psychologists, or the duration of such sessions. Similarly, no information was collected regarding the use of external tinnitus-related treatments during the study period, which could have influenced tinnitus severity, tinnitus-related distress, and comorbid symptoms such as depression and anxiety. Furthermore, the absence of usability and satisfaction measures also limit the interpretation of participants’ subjective experiences with the intervention. Additionally, no standardized measure of sleep quality was included, despite evidence that tinnitus frequently co-occurs with sleep disturbances (41).

Longer term changes in tinnitus are not well documented. One study found that full remission is a rare, but tinnitus symptoms may decrease over time while quality of life and depression seem rather stable (42). However, research has suggested that treatment effects of ICBT for tinnitus are maintained one year after completing the intervention (43) and possibly even at 6-year follow-up (44).

In conclusion, this study evaluated the effectiveness of an internet-based treatment consisting of TRT and CBT had primary on tinnitus, and secondary on depression, anxiety, hyperacusis and misophonia in 144 patients diagnosed with tinnitus. The study found significant improvement in symptoms of tinnitus, however the reduction in symptoms was less clear for the mild group. There were also significant reductions in symptoms of anxiety, depression and hyperacusis, but not for misophonia. Future research should explore the treatment format using a randomized controlled design, monitor patients’ time spent on the digital treatment, and use qualitative designs to explore in-depth patient experiences.

## Data Availability

The raw data supporting the conclusions of this article will be made available by the authors, without undue reservation.
