# Supplementary material for: Digital treatment for tinnitus combining tinnitus retraining therapy and cognitive behavioral therapy: a Norwegian naturalistic study
Source: Front Psychiatry. 2026 Sep 10;17:1868527. doi: 10.3389/fpsyt.2026.1868527 (PMC13601282; doi:10.3389/fpsyt.2026.1868527)
Supplement: Supplementary file 1 [file SupplementaryFile1.docx]

**Supplemental Table 1**. Correlations between outcome measures.

|  | 1 | 2 | 3 | 4 | 5 | 6 | 7 | 8 | 9 | 10 | 11 | 12 | 13 | 14 |
| --- | --- | --- | --- | --- | --- | --- | --- | --- | --- | --- | --- | --- | --- | --- |
| 1. THI_pre |  |  |  |  |  |  |  |  |  |  |  |  |  |  |
| 2. THI_90 | .69 |  |  |  |  |  |  |  |  |  |  |  |  |  |
| 3. THI_180 | .63 | .88 |  |  |  |  |  |  |  |  |  |  |  |  |
| 4. HQ_pre | .33 | .30 | .20 |  |  |  |  |  |  |  |  |  |  |  |
| 5. HQ_90 | .29 | .43 | .35 | .70 |  |  |  |  |  |  |  |  |  |  |
| 6. HQ_180 | .23 | .37 | .33 | .59 | .69 |  |  |  |  |  |  |  |  |  |
| 7. MQ_pre | .20 | .22 | .18 | .64 | .59 | .39 |  |  |  |  |  |  |  |  |
| 8. MQ_90 | .21 | .33 | .30 | .53 | .64 | .54 | .79 |  |  |  |  |  |  |  |
| 9. MQ_180 | .18 | .36 | .37 | .47 | .63 | .59 | .77 | .90 |  |  |  |  |  |  |
| 10. PHQ_pre | .54 | .48 | .43 | .44 | .30 | .26 | .44 | .31 | .32 |  |  |  |  |  |
| 11. PHQ_90 | .37 | .56 | .46 | .34 | .40 | .39 | .40 | .49 | .47 | .65 |  |  |  |  |
| 12. PHQ_180 | .31 | .52 | .54 | .35 | .40 | .40 | .44 | .44 | .51 | .67 | .80 |  |  |  |
| 13. GAD_pre | .56 | .48 | .47 | .44 | .35 | .30 | .49 | .41 | .45 | .76 | .54 | .52 |  |  |
| 14. GAD_90 | .37 | .56 | .49 | .38 | .45 | .40 | .44 | .55 | .54 | .57 | .76 | .66 | .72 |  |
| 15. GAD_180 | .38 | .57 | .58 | .29 | .36 | .46 | .37 | .49 | .58 | .54 | .66 | .74 | .69 | .80 |

*Note*. All correlations of .22 and above were significant at .01 level, while the correlations of .21 and below were significant at .05 level. Correlations between .00 and .29 are marked green, .30 to .69 in yellow, and .70+ in red. THI = Tinnitus Handicap Inventory; MQ = Misophonia Questionnaire; HQ = Hyperacusis Questionnaire; PHQ-9 = Patient Health Questionnaire-9; GAD-7 = General Anxiety Disorder-7; Pre = pre-treatment; 90 = 90-day assessment; 180 = 180-day assessment.
