# Supplementary material for: Digital treatment for tinnitus combining tinnitus retraining therapy and cognitive behavioral therapy: a Norwegian naturalistic study
Source: Front Psychiatry. 2026 Sep 10;17:1868527. doi: 10.3389/fpsyt.2026.1868527 (PMC13601282; doi:10.3389/fpsyt.2026.1868527)
Supplement: Supplementary file 2 [file SupplementaryFile2.docx]

Supplemental Figure 1. Screenshots of treatment elements.

*Note*. The treatment elements are originally in Norwegian, but the text has been translated into English for the purpose of this manuscript.
